# Clonal Diversity, Virulence Potential and Antimicrobial Resistance of *Escherichia coli* Causing Community Acquired Urinary Tract Infection in Switzerland

**DOI:** 10.3389/fmicb.2017.02334

**Published:** 2017-12-01

**Authors:** Magdalena T. Nüesch-Inderbinen, Melinda Baschera, Katrin Zurfluh, Herbert Hächler, Hansjakob Nüesch, Roger Stephan

**Affiliations:** ^1^National Centre for Enteropathogenic Bacteria and Listeria, Institute for Food Safety and Hygiene, University of Zurich, Zurich, Switzerland; ^2^Practice for General and Internal Medicine, Seuzach, Switzerland

**Keywords:** community acquired UTI, UPEC clones, ST141, virulence, *bla*_CTX−M_, *mph(A)*, resistance, fosfomycin

## Abstract

**Objectives:** The aim of this study was to assess the clonal structure, virulence potential and antibiotic susceptibility of uropathogenic *Escherichia coli* (UPEC) isolates causing community acquired urinary tract infection (CAUTI) in unselected primary care patients in Switzerland.

**Methods:** We performed multilocus sequence typing, virulence factor determination, and phenotypic and genotypic antimicrobial resistance testing on 44 non-duplicate UPEC isolates.

**Results:** Twenty-seven different sequence types (STs) were identified. Major UPEC clones were represented by 19 (43.2%) of the isolates, including *E. coli* ST131, ST69 (both 13.6%), ST73 (6.8%), ST10 (4.5%), ST127, ST140, (both 2.3%). Five (11.4%) isolates belonged to ST141.

Aggregate virulence factor (VF) scores were highest among isolates belonging to ST127 and ST141. Overall, 50% of the isolates were susceptible to all 12 antimicrobials tested, and all isolates remained susceptible to fosfomycin and nitrofurantoin. Resistance to sulfamethoxazole and ciprofloxacin were found in 31.8, and 15.9% of the isolates, respectively. Plasmid-mediated resistance genes were detected in ST69 and ST131 and included *aac(6*′*)-Ib-cr* (2.3% of all isolates) *bla*_CTX−M−14_ and *bla*_CTX−M−15_ (9%), and *mph(A)* (13.6%). None of the isolates tested positive for *mcr-1* or *mcr-2*.

**Conclusions:** Our results show that CAUTI in Switzerland is caused by a wide variety of UPEC STs for which fosfomycin remains a good treatment option. We suggest that ST141 is an emerging clone associated with UTI in the community, and warrants closer attention. Moreover, the high rate of *E. coli* harboring *mph(A)* from patients without a history of antimicrobial therapy or hospitalization indicates that UPEC is an important reservoir for *mph(A)*.

## Introduction

Urinary tract infection (UTI) is one of the most common bacterial infections, frequently affecting young women and women in the early postmenopausal period (Nicolle, 2013). UTI represent a major public health burden worldwide (Marrs et al., 2005; Totsika et al., 2012). *Escherichia coli* is the single most important causative agent of UTI, accounting for 75–85% of the episodes (Foxman, 2010). Although *E. coli* occurs naturally in the digestive tract of humans and animals as a commensal non-pathogenic, two subdivisions of this species constitute, by virtue of their possession of virulence factors (VF), etiological agents of either intestinal infections or extraintestinal diseases (Nataro and Kaper, 1998). Extraintestinal pathogenic *E. coli* (ExPEC) include uropathogenic *E. coli* (UPEC), newborn meningitis *E. coli* (NMEC), and avian pathogenic *E. coli* (APEC) which causes respiratory tract infections and septicemia in poultry (Kaper et al., 2004). Many UPEC strains belong to a limited number of clonal groups exhibiting specific genotypes, a range of virulence characteristics and varying patterns of resistance to antimicrobials (Gibreel et al., 2012). Pandemic UPEC clones include sequence type (ST)131, ST69, ST73, and ST95 (Tartof et al., 2005; Totsika et al., 2012; Banerjee et al., 2013a; Doumith et al., 2015). Other lineages such as ST10 and ST127 represent more recently emerged UPEC clones (Gibreel et al., 2012; Olesen et al., 2012; Alghoribi et al., 2014). A plethora of VF genes encoding adhesins, toxins, siderophores, capsules, and other factors have been associated with UPEC but to date, no specific set of virulence factors exist for defining an *E. coli* as uropathogenic (Flores-Mireles et al., 2015; Singer, 2015). Globally successful, highly virulent UPEC lineages are recognized for their potential to acquire and disseminate antimicrobial resistance genes, for example the extended-spectrum ß-lactamase (ESBL)-producing *E. coli* ST131 or the trimethoprim-sulfamethoxazole resistant *E. coli* Clonal Group A (CGA), a clone that clusters within ST69 (Woodford et al., 2011; Riley, 2014). Antimicrobial resistance affects every level of health care, including primary care (Goossens et al., 2005; European Centre for Disease Prevention and Control, European Food Safety Authority, and European Medicines Agency, 2017). However, information of the prevalence of resistance among *E. coli* causing community-acquired urinary tract infections (CAUTI) is rare. Treatment for uncomplicated CAUTI is for the most part empirical and does not include obtaining a urine specimen for culture. Diagnostic laboratory-based data on resistant UPEC thus represent a subset of *E. coli* causing CAUTI and furthermore do not include molecular characterization of the isolates. The UPEC strains analyzed in this study are particular in that they originate from a primary health care setting where microbiological diagnostics are in most cases not required or recommended (Gupta et al., 2011). Hence, any potential bias toward over-representation of complicated clinical situations that laboratory-based strain collections may present is avoided. The aim of this study was to evaluate the clonal distribution, virulence markers and resistance patterns of UPEC collected from patients consulting a general practitioner with symptoms of UTI.

## Materials and methods

### Specimen and clinical data collection

Urine samples were collected between February 2016 and June 2016 from a total of 96 patients presenting to their general practitioner with symptoms of UTI. The practice for general medicine is situated in a suburban community in the region of Zürich, Switzerland and has a catchment area of 10,000 people of all age groups, levels of education and professions. The patient collective is therefore representative for the average Swiss primary care patient. Informed consent was obtained from the participating patients and the study was approved by the local ethics committee of Zürich (BASEC-Nr.Req-2016-00374). Medical records were reviewed to obtain demographic and clinical data. Comorbidities were defined as one or more coexisting medical conditions that were additional to the diagnosis of UTI. Dipstick urinalysis was performed using Combur® urine test sticks (Roche, Rotkreuz, Switzerland). Specimens giving an elevated white cell count were collected using sterile screw-cap collection tubes containing boric acid, sodium formate and sodium borate as a preservative (Becton Dickinson, Allschwil, Switzerland). Thereafter, samples were diluted 1:1,000 in sterile 0.9% NaCl and 100 μl were streaked on UTI Brilliance agar (Oxoid, Pratteln, Switzerland) and on MacConkey agar (Becton Dickinson, Allschwil, Switzerland) used as growth controls. From plates corresponding to samples with a viable cell count of ≥10^4^ colony forming units (cfu)/mL, single colonies with morphological Gram-negative characteristics were subcultured on MacConkey agar following standard laboratory procedures guidelines. Isolates were subjected to species identification using API ID 32 E (bioMérieux). A total of 44 non-duplicate clinical isolates of *E. coli* from 44 patients with UTI were obtained for the study.

### Phylogenetic and multilocus sequence typing

DNA from *E. coli* isolates were subjected to triplex PCR targeting the *chuA* gene, the *yjaA* gene and an unspecified DNA fragment termed TspE4.C2, as described previously (Clermont et al., 2000). Isolates were classified as belonging to one of the four phylogenetic groups A, B1, B2, or D.

For multilocus sequence typing of *E. coli* isolates, internal fragments of the seven housekeeping genes (*adk, fumC, gyrB, icd, mdh, purA*, and *recA*) were amplified by PCR from DNA, as described by Wirth et al. (2006). Sequencing of the amplification products was performed by Microsynth (Balgach). Sequences were imported into the *E. coli* MLST database website (http://mlst.warwick.ac.uk/mlst/dbs/Ecoli) to determine MLST types. Alleles and STs that had not been previously described were designated new ST, but not assigned numerical designations, since whole-genome sequencing was not performed.

All ST131 isolates were further analyzed using CH typing based on *fumC* and *fimH* allele combination (Weissman et al., 2012). Isolates belonging to the ST131-CTX-M-15-H30-Rx sublineage were identified by detection of the specific amino acid alteration Gly723 → Ala within the allantoin transporter-encoding gene, *ybbW*, using PCR primers and conditions described previously (Banerjee et al., 2013b). Isolates typed ST10, ST69, ST73, ST140 (CC95), ST127, and ST131 were regarded as belonging to major UPEC clones.

### Virulence factor (VF) determination

All 44 isolates were screened for genetic markers of virulence associated with ExPEC by conventional PCR using primers and conditions described previously for *afa, papAH, papC, papEF, sfaS, fyuA, hlyA, iutA*, KpsMII, PAI, and *traT* (Johnson and Stell, 2000; Johnson et al., 2015), *vat* and *yfcV* (Spurbeck et al., 2012). The aggregate VF score was defined as the number of unique VF detected for each isolate, counting the PAI marker as one.

Strains that were negative for KpsMII were tested by PCR for the presence of the group 2 capsule subgroup K15 as described by Johnson et al. (2015).

Isolates that were positive for ≥2 of the five markers *afa, papAH*, and/or *papC, sfa*, KpsM II, or *iutA* were presumed to be ExPEC, and isolates that tested positive for ≥3 of the four genes *chuA, fyuA, vat*, or *yfcV* were considered UPEC, as described by Johnson et al. (2015).

Strains that tested negative for all VFs were screened by PCR for the presence of *aggR*, which encodes is a transcriptional regulator of enteroaggregative *E. coli* (EAEC) (Boisen et al., 2012).

### Phenotypic and genotypic antimicrobial resistance testing

Antimicrobial susceptibility testing was performed using the disk-diffusion method and the antibiotics ampicillin (AM), amoxicillin-clavulanic acid (AMC), cefotaxime (CTX), nalidixic acid (NA), ciprofloxacin (CIP), gentamicin (GM), kanamycin (K), streptomycin (S), sulfamethoxazole (SMZ), trimethoprim (TMP) tetracycline (T), and chloramphenicol (C) (Becton Dickinson, Heidelberg, Germany). Results were interpreted according to Clinical and Laboratory Standards Institute (CLSI) performance standards (Clinical Laboratory Standards Institute, 2016). For sulfamethoxazole, for which breakpoints are not listed separately from trimethoprim, an inhibition zone of ≤10 mm was interpreted as resistant. Isolates displaying resistance to three or more classes of antimicrobials (counting ß-lactams as one class) were defined as multidrug-resistant (MDR). Synergistic effects between AMC and CTX were regarded as an indication of the presence of an ESBL producer (Kaur et al., 2013). Putative ESBL producers were grown on Brilliance™ ESBL agar (Oxoid, Hampshire, UK). The presence of *bla*_ESBLs_ was confirmed by PCR and amplicons were sequenced as described previously (Pitout et al., 1998; Woodford et al., 2006; Geser et al., 2012; Zurfluh et al., 2015). Quinolone-resistant strains were examined for mutations in quinolone resistance-determining regions (QRDRs) of *gyrA* and *parC*, using previously described PCR and sequencing primers (Zurfluh et al., 2014). Screening for the plasmid-mediated fluoroquinolone resistance genes *aac(6*′*)-Ib-cr, qnrA, qnrB, qnrC, qnrD, qnrS*, and *qepA* genes was carried out as described previously (Park et al., 2006; Robicsek et al., 2006; Cattoir et al., 2007; Cavaco et al., 2009; Kim et al., 2009; Wang et al., 2009; Karczmarczyk et al., 2010; Zurfluh et al., 2014). Screening for the plasmid-mediated colistin resistance genes *mcr-1, mcr-2* and the plasmid-mediated azithromycin resistance gene *mph(A)* was performed using previously published primers (Ojo et al., 2004; Liassine et al., 2016; Liu et al., 2016) using appropriate positive controls (Nüesch-Inderbinen et al., 2016; Zurfluh et al., 2017).

Primers used in this study for targeting virulence and antimicrobial resistance genes are listed in Supplementary Table S1. Synthesis of primers and DNA custom sequencing was carried out by Microsynth (Balgach, Switzerland) and nucleotide sequences were analyzed with CLC Main Workbench 6.6.1. For database searches the BLASTN program of NCBI (http://www.ncbi.nlm.nih.gov/blast/) was used.

### Statistical analysis

Comparisons of proportions of *E. coli* STs, proportions of virulence genes and ExPEC/UPEC status within patient comorbidity status were performed by Fisher's exact test in a series of individual pairwise comparisons using 2 × 2 tables where each characteristic was determined as present or absent. The significance criterion was set at *p* ≤ 0.05. Calculations were performed using the VassarStats website for statistical computation (http://www.vassarstats.net).

## Results

### Demographic and clinical data

The 44 strains analyzed in this study were isolated from urine samples of 44 patients with a female/male ratio of 39/5, and a median age of 53. Ages ranged from 16 to 87 and were allocated to either group 1, 16–45 years (*n* = 18), or group 2, 46–87 years (*n* = 26), respectively (Table 1). A history of hospitalization during the 4 months before participation in the study was noted for 2 (4.5%) of the patients (Supplementary Table S2). None of the patients had a history of antimicrobial therapy in the 4 months before sample collection. Comorbidities were recorded for 17 (38.6%) of the patients (five in age group 1 and 12 in age group 2) and are summarized in Table 1 and detailed in Supplementary Table S2. Treatment following clinical diagnosis of UTI included fosfomycin (19/43.2% of the patients), norfloxacin (12/27.3%), ciprofloxacin (4/9%), or sulfamethoxazole/trimethoprim (3/6.8%). Six (13.6%) of the patients received no treatment (Supplementary Table S2).

**Table 1:** Demographic and clinical features of 44 UPEC belonging to major clonal complexes (CC) and to other sequence types (ST).

**Feature**	**Total (*n* = 44)**	**No. isolates by ST**							
		**Major UPEC lineages (*****n*** = **19)**						**Other STs (*****n*** = **25)**	
		**CC10 ST10 (*n* = 2)**	**CC69 ST69 (*n* = 6)**	**CC73 ST73 (*n* = 3)**	**CC95 ST140 (*n* = 1)**	**CC131 ST131 (*n* = 6)**	**ST127 (*n* = 1)**	**ST141 (*n* = 5)**	**Singly occurring STs^a^ (*n* = 20)**
**AGE GROUP**									
16–45 years	18	1	2	1	0	3	1	2	8
46–87 years	26	1	4	2	1	3	0	3	12
**GENDER**									
Female	39	2	6	1	1	5	1	5	18
Male	5	0	0	2	0	1	0	0	2
**COMORBIDITIES**									
Cancer	4	–	–	–	–	–	–	–	4
CVD	3	–	–	1	–	1	–	–	1
Diabetes	3	–	–	–	–	1	–	1	1
GID	2	–	–	–	–	–	–	–	2
MS	1	–	–	–	–	–	–	–	1
Polymorbidity	4	–	–	–	1	1	–	–	2
None	27	2	6	2	0	3	1	4	9

a *Singly occurring STs included ST12 (n = 1), ST14 (n = 1); ST1193 (n = 1); ST59 (n = 1), ST349, (n = 1), ST108 (n = 1), ST841 (n = 1), ST58 (n = 1), ST4628 (n = 1), ST2628 (n = 1), ST1851 (n = 1), ST40 (n = 1), ST720 (n = 1), ST681(n = 1), ST978 (n = 1), ST1001 (n = 1), ST4299 (n = 1), new STs (n = 3). The ST were not assigned numerical designations by the E. coli MLST database (http://mlst.warwick.ac.uk/mlst/dbs/Ecoli). CVD, cardiovascular diseases; GID, gastrointestinal diseases (irritable bowel syndrome and sigmoid diverticulitis, respectively); MS, multiple sclerosis; –, not observed*.

### Phylogenetic groups, STs and VF distribution

The majority (52.3%) of the 44 UPEC isolates belonged to phylogenetic group B2, followed by group D (22.7%), group A (13.6%) and B1 (11.4%). Twenty-seven different ST were identified, the four most common represented by ST131 (*n* = 6/13.6% of the isolates), ST69 (*n* = 6/13.6%), ST141 (*n* = 5/11.4%) and ST73 (*n* = 3/6.8%). Overall, 19 (43.2%) showed STs associated with major UPEC clonal groups (ST10, ST69, ST73, ST140, ST127, or ST131). Two (4.5%) belonged to ST12, and ST14, which are found occasionally among UPEC (Banerjee et al., 2013a; Nichols et al., 2016). Twenty (45.5%) of the isolates belonged to STs that occurred only once, whereof three were new STs. The new STs were not assigned numerical designations by the *E. coli* MLST database (http://mlst.warwick.ac.uk/mlst/dbs/Ecoli), however, the allelic profiles were related to those of ST23 (*n* = 2) and ST165 (*n* = 1) (Supplementary Table S3). Subtyping of the ST131 isolates identified five isolates with the *fumC*/*fimH* type 40-30, and one isolate with the *fumC*/*fimH* type 40–41 (Supplementary Table S2). Accordingly, the isolates were assigned to the subclones ST131-H30 and ST131-H41, respectively. All five ST131-H30 isolates were fluoroquinolone resistant and thus considered to belong to the subclone H30-R as determined by Price et al. (2013). Furthermore, both ST131-CTX-M-15 isolates were identified as belonging to the H30-Rx sublineage (Price et al., 2013).

The distribution of STs among the age groups, genders and comorbidities of the patients is shown in Table 1. ST distribution was invariable for age groups and gender, but varied by health status. The majority (*n* = 14/73.7%) of the 19 isolates that belonged to major UPEC lineages were isolated from patients without comorbidities, without reaching statistical significance (*p* = 0.2133). By contrast, the majority (*n* = 11/64.7%) of the 20 isolates that belonged to singly occurring STs were from patients for whom comorbidities were noted, approximating, but not reaching statistical significance (*p* = 0.0633).

Overall, 90.9% of the isolates tested positive for one or more of the VF genes (Table 2). Among the 44 isolates, the prevalence of individual VF genes ranged from 13.6% (*afa*) to 70.5% (*fyuA*). None of the KpsMII negative isolates were positive for K15 (data not shown). The overall median aggregate VF scores (and ranges) were lowest for isolates belonging to ST10 (median VF score 3.5, range 3–4) and highest for ST127 (median VF score 9, range 9) and ST141 (median VF score 11, range 5–12). Among the different STs, PAI, *yfcv* and *hlyA* were distinguished by their absence from ST69 (Table 2).

**Table 2:** Genotypic and phenotypic characteristics of 44 UPEC belonging to major clonal complexes (CC) or to other sequence types (ST).

**Feature**	**No. (%) of isolates by ST**								
	**Major UPEC clones**						**Other STs**		
	**CC10 ST10 (*n* = 2)**	**CC69 ST69 (*n* = 6)**	**CC73 ST73 (*n* = 3)**	**CC95 ST140 (*n* = 1)**	**ST127 (*n* = 1)**	**CC131 ST131 (*n* = 6)**	**ST141 (*n* = 5)**	**Singly occurring STs^a^ (*n* = 20)**	**Total (%)**
**VF GENES**									
*afa*	1 (50)	1 (16.7)	0 (0)	0 (0)	0 (0)	4 (66.7)	0 (0)	0 (0)	6 (13.6)
*papAH*	0 (0)	3 (50)	1 (33.3)	0 (0)	1 (100)	2 (33.3)	3 (60)	6 (30)	16 (36.4)
*papC*	0 (0)	3 (50)	1 (33.3)	0 (0)	0 (0)	2 (33.3)	3 (60)	6 (30)	15 (34)
*papEF*	0 (0)	4 (66.7)	1 (33.3)	0 (0)	1 (100)	2 (33.3)	3 (60)	5 (25)	16 (36.4)
*sfaS*	0 (0)	0 (0)	3 (100)	0 (0)	1 (100)	0 (0)	5 (100)	4 (20)	13 (29.5)
*yfcv*	1 (50)	0 (0)	3 (100)	1 (100)	1 (100)	4 (66.7)	5 (100)	8 (40)	23 (52.3)
*hlyA*	0 (0)	0 (0)	3 (100)	0 (0)	1 (100)	1 (16.6)	3 (60)	4 (20)	12 (27.3)
*vat*	0 (0)	0 (0)	3 (100)	1 (100)	1 (100)	0 (0)	4 (80)	6 (30)	15 (34)
*fyuA*	1 (50)	4 (66.7)	3 (100)	1 (100)	1 (100)	6 (100)	5 (100)	10 (50)	31 (70.5)
*iutA*	2 (100)	5 (83.3)	0 (0)	1 (100)	0 (0)	5 (83.3)	4 (80)	7 (35)	24 (54.5)
KpsMII	1 (50)	5 (83.3)	3 (100)	1 (100)	1 (100)	5 (83.3)	4 (80)	10 (50)	30 (68.2)
PAI	0 (0)	0 (0)	3 (100)	1 (100)	1 (100)	4 (66.7)	5 (100)	8 (40)	22 (50)
*traT*	1 (50)	5 (83.3)	0 (0)	0 (0)	0 (0)	4 (66.7)	2 (40)	8 (40)	20 (45.5)
VF score [mean, median, (range)]	3.5,3.5, (3–4)	5, 5, (2-7)	8, 7 (7–10)	6, 6, (6)	9, 9, (9)	6.5, 7, (5–7)	9.2, 11,(5–12)	4.1, 3.5 (0–11)	5.5, 5.5 (0–12)
**AMR PHENOTYPE**^b^									
Pansusceptible	2 (100)	1 (16.6)	3 (100)	1 (100)	0 (0)	0 (0)	2 (40)	13 (65)	22 (50)
Fosfomycin	0 (0)	0 (0)	0 (0)	0 (0)	0 (0)	0 (0)	0 (0)	0 (0)	0 (0)
Nitrofurantoin	0 (0)	0 (0)	0 (0)	0 (0)	0 (0)	0 (0)	0 (0)	0 (0)	0 (0)
Ciprofloxacin	0 (0)	1 (16.6)	0 (0)	0 (0)	0 (0)	5 (83.3)	0 (0)	1 (5)	7 (15.9)
Sulfamethoxazole	0 (0)	5 (83.3)	0 (0)	0 (0)	0 (0)	4 (66.7)	1 (20)	4 (20)	14 (31.8)
Trimethoprim	0 (0)	4 (66.7)	0 (0)	0 (0)	0 (0)	3 (50)	0 (0)	2 (10)	9 (20.5)
Ampicillin	0 (0)	4 (66.7)	0 (0)	0 (0)	0 (0)	5 (83.3)	2 (40)	3 (15)	14 (31.8)
Cefotaxime	0 (0)	1 (16.6)	0 (0)	0 (0)	0 (0)	3 (50)	0 (0)	0 (0)	4 (9)
MDR	0 (0)	4 (66.7)	0 (0)	0 (0)	0 (0)	5 (83.3)	2 (40)	2 (10)	13 (29.5)
**ACQUIRED AMR GENES**^c^									
*aac(6′)-Ib-cr*	0 (0)	0 (0)	0 (0)	0 (0)	0 (0)	1 (16.6)	0 (0)	0 (0)	1 (2.3)
*bla*_CTX−M−14_	0 (0)	0 (0)	0 (0)	0 (0)	0 (0)	1 (16.6)	0 (0)	0 (0)	1 (2.3)
*bla*_CTX−M−15_	0 (0)	1 (16.6)	0 (0)	0 (0)	0 (0)	2 (33.3)	0 (0)	0 (0)	3 (6.8)
*mph(A)*	0 (0)	4 (66.7)	0 (0)	0 (0)	0 (0)	2 (33.3)	0 (0)	0 (0)	6 (13.6)

a *Singly occurring ST included ST12 (n = 1), ST14 (n = 1); ST1193 (n = 1); ST59 (n = 1), ST349, (n = 1), ST108 (n = 1), ST841 (n = 1), ST58 (n = 1), ST4628 (n = 1), ST2628 (n = 1), ST1851 (n = 1), ST40 (n = 1), ST720 (n = 1), ST681 (n = 1), ST978 (n = 1), ST1001 (n = 1), ST4299 (n = 1), new ST (n = 3). The new STs were not assigned numerical designations by the E. coli MLST database (http://mlst.warwick.ac.uk/mlst/dbs/Ecoli)*.

b *The most important antimicrobials for UTI treatment, as well as ß-lactams are shown. A detailed overview of all resistance profiles is given in Supplementary Table S2*. *AMR, antimicrobial resistance; CC, clonal complex; MDR, multidrug resistance; ST, sequence type; UPEC, uropathogenic E. coli; VF, virulence factor*.

c *None of the isolates harbored the plasmid-mediated colistin resistance genes mcr-1 or mcr-2*.

Applying the criteria for ExPEC and UPEC described above, 27 (61.4%) of the isolates qualified as ExPEC, and 22 (50%) as UPEC (Supplementary Table 2).

No statistically significant associations were observed for STs, VFs, or ExPEC/UPEC status with comorbidities (data not shown).

Notably, four (9%) of the isolates (belonging to a new ST, to ST40, ST841, and ST1001, respectively), tested negative for all ExPEC VF (Supplementary Table S2). Screening of these isolates for *aggR* remained negative throughout (data not shown).

### Antimicrobial susceptibility

Antimicrobial susceptibility rates for the *E. coli* isolates are summarized in Table 2 and shown in detail in Supplementary Table S2. Overall, 22 (50%) of the isolates remained susceptible to all antimicrobials tested. None of the isolates were resistant to fosfomycin or to nitrofurantoin. Isolates belonging to ST131 were the most extensively resistant, exhibiting a high prevalence of 83.3% of MDR, ciprofloxacin resistance, and ampicillin resistance. ST69 was characterized by a prevalence of 66.7% of MDR, trimethoprim and ampicillin resistance as well as a high rate of 83.3% resistance to sulfamethoxazole (Table 2). Overall, 10 (22.7%) of the isolates were resistant to nalidixic acid, and seven thereof were ciprofloxacin resistant (Table 2). Among the isolates displaying ciprofloxacin resistance, all revealed chromosomal mutations leading to amino acid substitutions in GyrA and ParC (Table 3). Only one of the ciprofloxacin resistant ST131 strains harbored the *aac(6*′*)-Ib-cr* gene. Further, one (16.7% of all *E. coli* ST69) and three (50%) of the ST131 isolates were ESBL producers and resistant to cefotaxime. These ESBLs were CTX-M-14 (*n* = 1) and CTX-M-15 (*n* = 3) (Table 2). Of note was the association of the plasmid-mediated azithromycin resistance gene *mph(A)* with ST69 and ST131.

**Table 3:** Amino acid substitutions in the QRDR of ciprofloxacin resistant UPEC isolates.

	**QRDR**			
	***gyrA***		***parC***	
***E. coli* ST (*n*)**	**Ser83 → Leu**	**Asp87 → Asn**	**Ser80 → Ile**	**Glu84 → Val**
	**(%)**	**(%)**	**(%)**	**(%)**
69 (1)	1 (100)	1 (100)	1 (100)	0 (0)
131 (5)	5 (100)	5 (100)	5 (100)	5 (100)
1193 (1)	1 (100)	1 (100)	1 (100)	0 (0)

*Asn, asparagine; Asp, aspartic acid; Glu, glutamic acid; gyrA: DNA gyrase (type II topoisomerase) gene; Ile, isoleucine; Leu, leucine; parC, topoisomerase IV gene; QRDR, quinolone resistance determining region; Ser, serine; ST, sequence type; UPEC, uropathogenic E. coli; Val, valine*.

## Discussion

In this study, we characterized the clonal diversity, virulence potential and antimicrobial resistance characteristics of UPEC obtained from unselected patients attending a general practitioner between February and October 2016 in Switzerland. Such isolates are not usually available, since the current practice of treatment does not involve obtaining a urine specimen for culture, except in the case of more severe infection.

The UPEC population was characterized by a diversity of clonal lineages and sequence types. Although major UPEC lineages ST10, ST69, ST73, ST140 (belonging to CC95), ST127 and ST131 were detected in 43.2% of the cases, our results show that the majority (56.8%) of *E. coli* causing UTI in primary care patients in this particular setting belongs to other STs, irrespective of the patients' health status.

Among the major UPEC lineages, we observed ST131 isolates belonging to two different subclones, H30-R and H41, respectively. Further, we detected non-ESBL and ESBL-producers among the ST131-H30-R strains, including CTX-M-14 and CTX-M-15 producing strains. The prevalence of *E. coli* ST131-CTX-M-15-H30-Rx among primary care UPEC isolates in this study was 4.5%. Currently, available studies with which to compare our results are scarce. In a previous study on clinical ESBL producing *E. coli* isolates in Switzerland, Seiffert et al. (2013) observed fluoroquinolone resistant CTX-M-15 producing *E. coli* ST131 in two of 95 (2.1%) community-associated UTI. However, lack of data on the clonal background of these earlier strains limits a direct comparison and highlights the need of further studies to assess the prevalence of ST131-H30-Rx among UPEC isolates in Switzerland. Our findings suggest that multiple subclones of ST131 are circulating in the community, likely as intestinal commensals, as suggested previously (Leflon-Guibout et al., 2008). Moreover, this study shows that *E. coli* ST131 and ST69 are disseminators of the plasmid-mediated azithromycin resistance gene *mph(A)* and that this gene occurs in isolates in the absence of any apparent selective pressure such as history of antimicrobial therapy or hospitalization of the patient. The dissemination of resistance to macrolides represents a threat to the treatment of infections caused by *Salmonella* and *Shigella* spp. and warrants further attention (Phuc Nguyen et al., 2009; Baker et al., 2015).

Interestingly, only one (2.1%) of the isolates (ST140) in this study belonged to CC95. This is similar to previous observations for community and hospital derived UPEC isolates from the United Kingdom and from Germany (Croxall et al., 2011; Toval et al., 2014). By contrast, this clone is more prevalent among UTI in the U.S.A. and Australia (Banerjee et al., 2013a; Gordon et al., 2017). Therefore, although CC95 strains occur globally, their distribution is unequal across continents and countries. Our findings suggest that the uncomplicated CAUTI may not commonly caused *E. coli* CC95 in this particular geographical setting.

Some other STs identified in this study are infrequently associated with UPEC, including ST10, ST12, and ST14, (Banerjee et al., 2013a; Nichols et al., 2016), and ST141 (Toval et al., 2014; Leatham-Jensen et al., 2016). ST141 has also been identified in the fecal flora of healthy individuals (Leflon-Guibout et al., 2008). The results from this study suggest that ST141 has emerged as a UPEC clone in the community in Switzerland.

A substantial percentage (38.6 and 50%, respectively) of the isolates did not meet the ExPEC or UPEC criteria defined for this study. Moreover, four (9%) of the isolates did not reveal any ExPEC or UPEC associated VF and in addition remained susceptible to all antimicrobials tested. Since UTI is sporadically caused by EAEC (Herzog et al., 2014; Lara et al., 2017), these four isolates were screened for the EAEC marker gene *aggR*. However, all remained negative and the virulence mechanisms of these UPEC isolates remain to be further characterized. These observations are supportive of a concept involving a broader definition of the ability of *E. coli* to cause uncomplicated CAUTI in humans with or without comorbidities, such as bacterial transcriptional regulation and host susceptibility (Schreiber et al., 2017). Nonetheless, although the panel of VFs examined included acknowledged UPEC associated genes, it is limited in number and is composed of only a subset of known VFs. Other important determinants of UPEC may have been overlooked, including minor sequence variants of known VFs and unrecognized VFs. Further limitations of this study is the small sample size which could have compromised statistical power, consequently introducing the possibility of type II errors, i.e., failure to detect a difference when one actually exists. Finally, this study was conducted with samples originating from one particular practice for general medicine in a single suburban setting. Caution should be exercised in making generalizations across different populations, countries and settings.

Treatment for UTI at community level in Switzerland as in other countries, is for the most part empirical and follows the recommendations of the Infectious Diseases Society of America (IDSA) and the European Society for Microbiology and Infectious Diseases (ESCMID) (Gupta et al., 2011). Recommendations include fosfomycin, sulfamethoxazole/trimethoprim (SMZ/TMP), nitrofurantoin, and pevmecillinam, with fluoroquinolones approved as an important alternative agent. A resistance prevalence of 20% is considered the threshold at which an antimicrobial is no longer recommended. In this study, 31.8% of the UPEC isolates were resistant to SMZ, and 15.9% to CIP. Therefore, the use of SMZ/TMP for the empirical treatment of UTI due to UPEC at primary care level in this region is questionable, and the safety of treatment with fluoroquinolones is threatened. By contrast, fosfomycin remains a valuable antimicrobial for treating CAUTI due to *E. coli*, including infections caused by pandemic, highly virulent MDR UPEC clones.

In conclusion, we found that UTI in primary care patients in the study community in Switzerland is caused by a diversity of *E. coli* STs, including, but not predominantly, pandemic clones such as ST131, ST69. *E. coli* ST141 was identified as an emerging clone that is associated with UTI in the community, and warrants closer attention. Antimicrobial resistance and carriage of plasmid-mediated *bla*_CTX−M_ and *mph(A)* is linked to ST131 including its globally disseminated sublineage H30-Rx, and ST69. Our results indicate that SMZ/TMP and fluoroquinolone resistance in UPEC in the primary care setting may compromise the treatment of UTI. By contrast, fosfomycin remains an optimal first line antimicrobial for the treatment of UTI at primary care level.

## Author contributions

MN-I, HH, HN, and RS: designed the study; MB and KZ: carried out the microbiological and molecular biological tests; MN-I, MB, and RS: analyzed and interpreted the data; MN-I drafted the manuscript. All authors read and approved the final manuscript.

## Conflict of interest statement

The authors declare that the research was conducted in the absence of any commercial or financial relationships that could be construed as a potential conflict of interest.
