# Supplementary material for: Clonal Diversity, Virulence Potential and Antimicrobial Resistance of Escherichia coli Causing Community Acquired Urinary Tract Infection in Switzerland
Source: Front Microbiol. 2017 Dec 1;8:2334. doi: 10.3389/fmicb.2017.02334 (PMC5716990; doi:10.3389/fmicb.2017.02334)
Supplement: Supplementary file 1 [file Table1.docx]

***Supplementary Material***

**Clonal Diversity, Virulence Potential and Antimicrobial Resistance of *Escherichia coli* causing Community Acquired Urinary Tract Infection in Switzerland**

Magdalena Nüesch-Inderbinen, Melinda Baschera, Katrin Zurfluh, Herbert Hächler, Hansjakob Nüesch, and Roger Stephan*

*** Correspondence:** stephanr@fsafety.uzh.ch

# Supplementary Table

Supplementary Table S1: PCR primers used in this study for targeting virulence and plasmid-mediated antimicrobial resistance genes

| **Primer ID** | **Nucleotide sequence (5'–3')** | **Target** | **Amplicon**  **(bp)** | **Annealing**  **temperature (°C)** | **References** |
| --- | --- | --- | --- | --- | --- |
| **Detection primers for virulence genes** | |  |  |  |  |
|  | |  |  |  |  |
| afaf | GGCAGAGGGCCGGCAACAGGC | *afa* | 594 | 63 | (Johnson et al., 2015) |
| afar | CCCGTAACGCGCCAGCATCTC | *afa* |  |  | (Johnson et al., 2015) |
| aggRf | GCAATCAGATTAARCAGCGATACA | *aggR* | 426 | 57 | (Boisen et al., 2012) |
| aggRr | CATTCTTGATTGCATAAGGATCTGG | *aggR* |  |  | (Boisen et al., 2012) |
| FyuA f | tgattaaccccgcgacgggaa | *fyuA* | 880 | 63 | (Johnson and Stell, 2000) |
| FyuA r | cgcagtaggcacgatgttgta | *fyuA* |  |  | (Johnson and Stell, 2000) |
| hly f | aacaaggataagcactgttctggct | *hlyA* | 1177 | 63 | (Johnson and Stell, 2000) |
| hly r | accatataagcggtcattcccgtca | *hlyA* |  |  | (Johnson and Stell, 2000) |
| iutAf | GGCTGGACATCATGGGAACTGG | *iutA* | 302 | 63 | (Johnson et al., 2015) |
| iutAf | CGTCGGGAACGGGTAGAATCG | *iutA* |  |  | (Johnson et al., 2015) |

Supplementary Table 1 continued

| **Primer ID** | **Nucleotide sequence (5'–3')** | **Target** | **Amplicon**  **(bp)** | **Annealing**  **temperature (°C)** | **References** |
| --- | --- | --- | --- | --- | --- |
| **Detection primers for virulence genes** | |  |  |  |  |
|  |  |  |  |  |  |
| K15 f | ACGGATTCACGACAAAGCTC | K15 | 581 | 63 | (Johnson et al., 2015) |
| K15 r | GGCAAATATCGCTTGGGTTA | K15 |  |  | (Johnson et al., 2015) |
| kii | GCGCATTTGCTGATACTGTTG | Kps II | 570 | 63 | (Johnson et al., 2015) |
| KpsII | AGGTAGTTCAGACTCACACCT | Kps II |  |  | (Johnson et al., 2015) |
| PapA f | atggcagtggtgtcttttggtg | *papAH* | 720 |  | (Johnson and Stell, 2000) |
| PapA r | cgtcccaccatacgtgctcttc | *papAH* |  |  | (Johnson and Stell, 2000) |
| PapC f | gtggcagtatgagtaatgaccgtta | *papC* | 200 |  | (Johnson and Stell, 2000) |
| PapC r | atatcctttctgcagggatgcaata | *papC* |  |  | (Johnson and Stell, 2000) |
| PapEF f | gcaacagcaacgctggttgcatcat | *papEF* | 336 |  | (Johnson and Stell, 2000) |
| PapEF r | agagagagccactcttatacggaca | *papEF* |  |  | (Johnson and Stell, 2000) |
| RPAi f | ggacatcctgttacagcgcgca | PAI | 930 | 63 | (Johnson and Stell, 2000) |
| RPAi r | tcgccaccaatcacagccgaac | PAI |  |  | (Johnson and Stell, 2000) |
| sfaSf | GTGGATACGACGATTACTGTG | *sfaS* | 244 | 63 | (Johnson et al., 2015) |
| sfaSr | CCGCCAGCATTCCCTGTATTC | *sfaS* |  |  | (Johnson et al., 2015) |
| TraT f | ggtgtggtgcgatgagcacag | *traT* | 290 | 63 | (Johnson and Stell, 2000) |
| TraT r | cacggttcagccatccctgag | *traT* |  |  | (Johnson and Stell, 2000) |
| Vat f | TCAGGACACGTTCAGGCATTCAGT | *vat* | 1100 | 63 | (Spurbeck et al., 2012) |
| Vat r | GGCCAGAACATTTGCTCCCTTGTT | *vat* |  |  | (Spurbeck et al., 2012) |
| yfcvf | ACATGGAGACCACGTTCACC | *yfcv* | 292 | 63 | (Spurbeck et al., 2012) |
| yfcvr | GTAATCTGGAATGTGGTCAGG | *yfcv* |  |  | (Spurbeck et al., 2012) |
|  | |  |  |  |  |
| **Detection primers for antimicrobial resistance genes** | |  |  |  |  |
|  | |  |  |  |  |
| 2_1_ forward | AAAAATCACTGCGCCAGTTC | *bla*_CTX-M group1_ | 415 | 52 | (Woodford et al., 2006) |
| 3_1_ reverse | AGCTTATTCATCGCCACGTT | *bla*_CTX-M group1_ |  | 52 | (Woodford et al., 2006) |
| 2_2_ forward | CGACGCTACCCCTGCTATT | *bla*_CTX-M group2_ | 552 | 52 | (Woodford et al., 2006) |
| 3_2_ reverse | CCAGCGTCAGATTTTTCAGG | *bla*_CTX-M group2_ |  | 52 | (Woodford et al., 2006) |
| 2_9_ forward | CAAAGAGAGTGCAACGGATG | *bla*_CTX-M group9_ | 209 | 52 | (Woodford et al., 2006) |
| 3_9_ reverse | ATTGGAAAGCGTTCATCACC | *bla*_CTX-M group9_ |  | 52 | (Woodford et al., 2006) |
| Gr. 8 CTX-M-fw | ATG AGA CAT CGC GTT AAG CGG ATG | *bla_CTX-M-_*_group8_ | 829 | 60 | (Zurfluh et al., 2015) |
| Gr. 8 CTX-M-rev | CAC GAC GAC TTT CTG CCT TCT GC | *bla_CTX-M-_*_group8_ |  |  | (Zurfluh et al., 2015) |
| A | CACTCAAGGATGTATTGTG | *bla*_SHV_ | 885 | 50 | (Pitout et al., 1998) |
| B | TTAGCGTTGCCAGTGCTCG | *bla*_SHV_ |  |  | (Pitout et al., 1998) |

Supplementary Table 1 continued

| **Primer ID** | **Nucleotide sequence (5'–3')** | **Target** | | **Amplicon**  **(bp)** | **Annealing**  **temperature (°C)** | **References** |
| --- | --- | --- | --- | --- | --- | --- |
| **Detection primers for antimicrobial resistance genes** | |  |  |  |  |  |
|  |  |  |  |  |  |  |
| aac(6')-Ib_For | TTGCGATGCTCTATGAGTGGCTA | *aac(69)-Ib-cr* | | 482 | 55 | (Park CH, 2006) |
| aac(6')-Ib_Rev | CTCGAATGCCTGGCGTGTTT | *aac(69)-Ib-cr* | |  |  | (Park CH, 2006) |
| C | TCGGGGAAATGTGCGCG | *bla*_TEM_ | | 971 | 50 | (Pitout et al., 1998) |
| D | TGCTTAATCAGTGAGGCACC | *bla*_TEM_ | |  |  | (Pitout et al., 1998) |
| CLR5-F | CGGTCAGTCCGTTTGTTC | *mcr-1* | | 309 | 57 | (Liu et al., 2016) |
| CLR5-R | CTTGGTCGGTCTGTA GGG | *mcr-1* | |  |  | (Liu et al., 2016) |
| mcr-2 full Fw | ATGACATCACATCACTCTTGG | *mcr-2* | | 567 | 65 | (Liassine et al., 2016) |
| mcr-2 full Rv | TTACTGGATAAATGCCGCGC | *mcr-2* | |  |  | (Liassine et al., 2016) |
| mphAF | GTG AGG AGG AGC TTC GCG AG | *mph(A)* | | 403 | 60 | (Ojo et al., 2004) |
| mphAR | TGC CGC AGG ACT CGG AGG TC | *mph(A)* | |  |  | (Ojo et al., 2004) |
| QEPfor | TGGTCTACGCCATGGACCTCA | *qepA* | | 1137 | 56 | (Karczmarczyk et al., 2010) |
| QEPrev | TGAATTCGGACACCGTCTCCG | *qepA* | |  |  | (Karczmarczyk et al., 2010) |
| QnrAm-F | AGAGGATTTCTCACGCCAGG | *qnrA* | | 516 | 54 | (Cattoir et al., 2007) |
| qnrA_R | GCCATACCTACGGCGATACC | *qnrA* | |  |  | (Robicsek et al., 2006) |
| qnrB_F | GATCGTGAAAGCCAGAAAGG | *qnrB* | | 476 | 54 | (Kim et al., 2009) |
| qnrB_R | ATGAGCAACGATGCCTGGTA | *qnrB* | |  |  | (Kim et al., 2009) |
| qnrC-F | GGGTTGTACATTTATTGAATC | *qnrC* | | 447 | 47 | (Wang et al., 2009) |
| qnrC-R | TCCACTTTACGAGGTTCT | *qnrC* | |  |  | (Wang et al., 2009) |
| qnrD fw | CGAGATCAATTTACGGGGAATA | *qnrD* | | 582 | 54 | (Cavaco et al., 2009) |
| qnrD rev | AACAAGCTGAAGCGCCTG | *qnrD* | |  |  | (Cavaco et al., 2009) |
| QnrSm-F | GCAAGTTCATTGAACAGGGT | *qnrS* | | 428 | 54 | (Cattoir et al., 2007) |
| QnrSm-R | TCTAAACCGTCGAGTTCGGCG | *qnrS* | |  |  | (Cattoir et al., 2007) |
|  |  |  | |  |  |  |
| **Amplifying and sequencing primers** | |  | |  |  |  |
|  |  |  | |  |  |  |
| 1_1_ forward | AAACACACGTGGAATTTAGGG | *bla*_CTX-M group1_ | | 1097 | 52 | (Geser et al., 2012) |
| 4_1_ reverse | CCGTCGGTGACGATTTTAGCC | *bla*_CTX-M group1_ | | 865 |  | (Geser et al., 2012) |
| 5_1_ reverse | CCGATGACTATGCGCACTGGG | *bla*_CTX-M group1_ | |  |  | (Geser et al., 2012) |
| 1_2_ forward | TTTTGCCGTACCTGCGTACCC | *bla*_CTX-M group2_ | | 1187 | 52 | (Geser et al., 2012) |
| 4_2_ reverse | CCGTGGGTTACGATTTTCGCC | *bla*_CTX-M group2_ | | 825 |  | (Geser et al., 2012) |
| 5_2_ reverse | TTGGTCCAGAAAAAAGAGCGG | *bla*_CTX-M group2_ | |  |  | (Geser et al., 2012) |
| 1_9_ forward | TGATGTAACACGGATTGACCG | *bla*_CTX-M group9_ | | 1061 | 52 | (Geser et al., 2012) |
| 4_9_ reverse | AAACCAGTTACAGCCCTTCGG | *bla*_CTX-M group9_ | | 866 |  | (Geser et al., 2012) |
| 5_9_ reverse | TGGAGCCACGGTTGATGAGGG | *bla*_CTX-M group9_ | |  |  | (Geser et al., 2012) |

Supplementary Table 1 continued

| **Primer ID** | | **Nucleotide sequence (5'–3')** | | **Target** | **Amplicon**  **(bp)** | **Annealing**  **temperature (°C)** | **References** |
| --- | --- | --- | --- | --- | --- | --- | --- |
| **Amplifying and sequencing primers** | | | |  |  |  |  |
|  | |  | |  |  |  |  |
| Gr. 8 CTX-M-fw | ATG AGA CAT CGC GTT AAG CGG ATG | | *bla_CTX-M-_*_group8_ | | 829 | 60 | (Zurfluh et al., 2015) |
| Gr. 8 CTX-M-rev | CAC GAC GAC TTT CTG CCT TCT GC | | *bla_CTX-M-_*_group8_ | |  |  | (Zurfluh et al., 2015) |
| gyrA WF | | AAATCTGCCCGTGTCGTTGGT | | *gyrA* | 344 | 55 | (Kim et al., 2009) |
| gyrA WR | | GCCATACCTACGGCGATACC | | *gyrA* |  |  | (Kim et al., 2009) |
| parC WF | | CTGAATGCCAGCGCCAAATT | | *parC* | 168 | 55 | (Kim et al., 2009) |
| parC WR | | GCGAACGATTTCGGATCGTC | | *parC* |  |  | (Kim et al., 2009) |
