# Supplementary material for: Clonal Diversity, Virulence Potential and Antimicrobial Resistance of Escherichia coli Causing Community Acquired Urinary Tract Infection in Switzerland
Source: Front Microbiol. 2017 Dec 1;8:2334. doi: 10.3389/fmicb.2017.02334 (PMC5716990; doi:10.3389/fmicb.2017.02334)
Supplement: Supplementary file 3 [file Table3.docx]

***Supplementary Material***

**Clonal Diversity, Virulence Potential and Antimicrobial Resistance of *Escherichia coli* causing Community Acquired Urinary Tract Infection in Switzerland**

Magdalena Nüesch-Inderbinen, Melinda Baschera, Katrin Zurfluh, Herbert Hächler, Hansjakob Nüesch, and Roger Stephan*

*** Correspondence**: stephanr@fsafety.uzh.ch

^*^

Table S3. Results of the MLST analysis for *E. coli* strains with new STs.^a^

| **Isolate ID** | **PG** |  | **MLST alleles** | | | | | | |  | **ST** | **Related ST(CC)** |
| --- | --- | --- | --- | --- | --- | --- | --- | --- | --- | --- | --- | --- |
|  |  |  | *adk* | *fumC* | *gyrB* | *icd* | *mdh* | *purA* | *recA* |  |  |  |
| PRAX-106 | A |  | 6 | 4 | 12 | 1 | 20 | 12 | 25 |  | New | ST88 (CC23) |
| PRAX-135 | A |  | 10 | 27 | 5 | 10 | 12 | 8 | 369 |  | New | ST165; ST189 (CC165) |
| PRAX-143 | A |  | 178 | 4 | 12 | 1 | 20 | 13 | 7 |  | New | ST461 (CC23) |

^a^The ST were not assigned numerical designations by the *E. coli* MLST database (http://mlst.warwick.ac.uk/mlst/dbs/Ecoli).

CC, clonal complex; MLST, multilocus sequence type; PG, phylogenetic group; ST, sequence type.
